# Deletions at 22q11.2 in idiopathic Parkinson's disease: a combined analysis of genome-wide association data

**DOI:** 10.1016/S1474-4422(16)00071-5

**Published:** 2016-05

**Authors:** Kin Y Mok, Una Sheerin, Javier Simón-Sánchez, Afnan Salaka, Lucy Chester, Valentina Escott-Price, Kiran Mantripragada, Karen M Doherty, Alastair J Noyce, Niccolo E Mencacci, Steven J Lubbe, International Parkinson's Disease Genomics Consortium (IPDGC), Caroline H Williams-Gray, Roger A Barker, Karin D van Dijk, Henk W Berendse, Peter Heutink, Jean-Christophe Corvol, Florence Cormier, Suzanne Lesage, Alexis Brice, Kathrin Brockmann, Claudia Schulte, Thomas Gasser, Thomas Foltynie, Patricia Limousin, Karen E Morrison, Carl E Clarke, Stephen Sawcer, Tom T Warner, Andrew J Lees, Huw R Morris, Mike A Nalls, Andrew B Singleton, John Hardy, Andrey Y Abramov, Vincent Plagnol, Nigel M Williams, Nicholas W Wood

**Affiliations:** a Department of Molecular Neuroscience, UCL Institute of Neurology, London, UK; b Reta Lila Weston Institute for Neurological Studies, UCL Institute of Neurology, London, UK; c Sobell Department of Motor Neuroscience, UCL Institute of Neurology, London, UK; d Department of Clinical Neuroscience, UCL Institute of Neurology, London, UK; e Division of Life Science, Hong Kong University of Science and Technology, Hong Kong Special Administrative Region, China; f Genetics and Epigenetics of Neurodegeneration, Hertie Institute for Clinical Brain Research (HIH), Tübingen, Germany; g Department of Neurodegenerative Diseases, Hertie Institute for Clinical Brain Research (HIH), Tübingen, Germany; h Genetics and Epigenetics of Neurodegeneration, Tübingen, Germany; i Genome Biology of Neurodegenerative Diseases, Tübingen, Germany; j German Center for Neurodegenerative Diseases (DZNE), Tübingen, Germany; k Institute of Psychological Medicine and Clinical Neurosciences, MRC Centre for Neuropsychiatric Genetics and Genomics, Cardiff University School of Medicine, Cardiff University, Cardiff, UK; l Medical Genetics and Laboratory Medicine Department, Faculty of Applied Medical Sciences, Umm Al-Qura University, Makkah, Saudi Arabia; m Department of Regional Neurosciences, Royal Victoria Hospital, Belfast, UK; n John van Geest Centre for Brain Repair, Department of Clinical Neurosciences, Forvie Site, University of Cambridge, Cambridge, UK; o Department of Clinical Neurosciences, Cambridge Biomedical Campus, University of Cambridge, Cambridge, UK; p Department of Neurology, Neuroscience Campus Amsterdam, VU University Medical Center, Amsterdam, Netherlands; q Research Unit U1127 at INSERM, Research Unit UMR 7225 at the French National Center for Scientific Research (CNRS), and Research Unit UMR_S 1127 at Pierre and Marie Curie University University of Paris VI at Sorbonne Universités, Institut du Cerveau et de la Moelle épinière Brain and Spine Institute (ICM), Paris, France; r Clinical Investigation Center Unit 1422 at INSERM and AP-HP Hôpital de la Pitié Salpêtrière, Centre d'Investigation Clinique Pitié Neurosciences, Paris, France; s Department of Neurodegenerative Diseases, University of Tübingen, Tübingen, Germany; t Institute of Clinical Sciences, College of Medical and Dental Sciences, University of Birmingham, Birmingham, UK; u School of Clinical and Experimental Medicine, University of Birmingham, Birmingham, UK; v Laboratory of Neurogenetics, National Institute on Aging, Bethesda, MD, USA; w UCL Genetics Institute, London, UK

## Abstract

**Background:**

Parkinson's disease has been reported in a small number of patients with chromosome 22q11.2 deletion syndrome. In this study, we screened a series of large, independent Parkinson's disease case-control studies for deletions at 22q11.2.

**Methods:**

We used data on deletions spanning the 22q11.2 locus from four independent case-control Parkinson's disease studies (UK Wellcome Trust Case Control Consortium 2, Dutch Parkinson's Disease Genetics Consortium, US National Institute on Aging, and International Parkinson's Disease Genomics Consortium studies), which were independent of the original reports of chromosome 22q11.2 deletion syndrome. We did case-control association analysis to compare the proportion of 22q11.2 deletions found, using the Fisher's exact test for the independent case-control studies and the Mantel-Haenszel test for the meta-analyses. We retrieved clinical details of patients with Parkinson's disease who had 22q11.2 deletions from the medical records of these patients.

**Findings:**

We included array-based copy number variation data from 9387 patients with Parkinson's disease and 13 863 controls. Eight patients with Parkinson's disease and none of the controls had 22q11.2 deletions (p=0·00082). In the 8451 patients for whom age at onset data were available, deletions at 22q11.2 were associated with Parkinson's disease age at onset (Mann-Whitney *U* test p=0·001). Age at onset of Parkinson's disease was lower in patients carrying a 22q11.2 deletion (median 37 years, 95% CI 32·0–55·5; mean 42·1 years [SD 11·9]) than in those who did not carry a deletion (median 61 years, 95% CI 60·5–61·0; mean 60·3 years [SD 12·8]). A 22q11.2 deletion was present in more patients with early-onset (p<0·0001) and late-onset Parkinson's disease (p=0·016) than in controls, and in more patients with early-onset than late-onset Parkinson's disease (p=0·005).

**Interpretation:**

Clinicians should be alert to the possibility of 22q11.2 deletions in patients with Parkinson's disease who have early presentation or features associated with the chromosome 22q11.2 deletion syndrome, or both.

**Funding:**

UK Medical Research Council, UK Wellcome Trust, Parkinson's UK, Patrick Berthoud Trust, National Institutes of Health, “Investissements d'Avenir” ANR-10-IAIHU-06, Dutch Parkinson Foundation (Parkinson Vereniging), Neuroscience Campus Amsterdam, National Institute for Health Research, National Institute on Aging, National Institutes of Health.

## Introduction

Parkinson's disease is the second most common neurodegenerative disease and is characterised by bradykinesia with resting tremor, stiffness, and gait disturbance, and a range of non-motor features. It involves the aggregation of α-synuclein as Lewy bodies and Lewy neurites in many motor and non-motor brain areas. The population prevalence is estimated at 0·3%, but increases with age, rising to 1–2% in people older than 60 years and 3–4% in those older than 80 years.^1^ The median age of onset is around 60 years.^2^

The identification of rare highly penetrant mutations in genes causing familial Parkinson's disease has substantially improved our understanding of the pathogenesis of this complex and common disorder.^2^ Simultaneously, our understanding of the idiopathic form of the disease has been enhanced by findings from large genome-wide association studies.^3^, ^4^, ^5^, ^6^, ^7^, ^8^

Microdeletions at chromosome 22q11.2 are the most frequent known interstitial deletions found in human beings, occurring in about 25 in 100 000 livebirths.^9^ These well-characterised deletions are inherited from an affected parent in 5–10% of cases and occur de novo in the remainder.^9^ About 87% of deletions span a shared 3 Mb region that includes at least 52 known genes, whereas around 8% span a smaller 1·5 Mb region (nested within the larger 3 Mb region) that contains at least 31 genes.^10^ The remainder also span the larger 3 Mb region, but with different breakpoints.^10^ The homogeneity of the deletion breakpoints is largely due to the presence of blocks of genomic sequence known as low copy repeats (LCRs) at the breakpoints of each deleted region.^10^ The LCRs are believed to act as targets for anomalous non-allelic homologous recombination during meiosis, thereby generating chromosomal rearrangements.

**Research in context**

**Evidence before this study**

Deletion at 22q11.2 is one of the most common deletions found in human beings and is one of the strongest identified genetic risk factors for schizophrenia. Findings from previous case reports and one case study have suggested a 22q11.2 deletion might be associated with Parkinson's disease. In comparison with studies of 22q11.2 deletions in schizophrenia, so far, no data from large Parkinson's disease studies exist regarding the potential role of 22q11.2 deletion on risk of Parkinson's disease. We screened data from independent genome-wide association studies to establish the frequency of 22q11.2 deletions in patients with Parkinson's disease.

**Added value of this study**

We provide evidence that deletions at 22q11.2 increase the risk of Parkinson's disease, particularly early-onset Parkinson's disease (onset age <45 years). An estimation of increased risk of early-onset Parkinson's disease, conferred by deletions at 22q11.2, is about a 20-times increase compared with the general population (assuming the proportion of carriers of a 22q11.2 deletion is 0·024%). This risk is comparable to the odds ratio for development of schizophrenia. The frequency of 22q11.2 deletion was significantly higher in Parkinson's disease overall (0·09%) than in controls, but was higher in early-onset (0·49%) than in late-onset (0·04%) Parkinson's disease. Presenting features of Parkinson's disease in 22q11.2 deletion carriers are similar to those in patients with Parkinson's disease in general, other than an earlier age of onset. Outcomes might be less favourable, with more rapid deterioration and mortality.

**Implications of all the available evidence**

Clinicians should bear this uncommon 22q11.2 deletion in mind when a patient presents with early-onset Parkinson's disease. Carriers of a 22q11.2 deletion are prone to many other potential comorbidities, and clinicians should actively investigate for these comorbidities when managing a patient with a 22q11.2 deletion and Parkinson's disease. Therefore, the 22q11.2 deletion links two different neuropsychiatric disorders: schizophrenia and Parkinson's disease. Whether there is a single common pathogenic pathway or whether separate genetic defects within the 22q11.2 deletion contribute to the differing phenotypes remains unclear. Nevertheless, a potential link through catechol-*O*-methyltransferase is plausible and should be investigated further.

Deletions at 22q11.2 have been associated with a heterogeneous range of clinical syndromes, which include DiGeorge syndrome and velocardiofacial syndrome and, as such, they are typically grouped together as chromosome 22q11.2 deletion syndrome. As would be expected from the product of this diverse range of clinical syndromes, the phenotype of chromosome 22q11.2 deletion syndrome is complex and typically includes psychiatric, craniofacial, immunological, and cardiovascular defects.

In 2013, by following up clinical case reports of four individuals who carried a hemizygous deletion at chromosome 22q11.2 and also had parkinsonism,^11^, ^12^, ^13^ Butcher and colleagues^14^ identified an additional four patients, one of whom had been reported previously,^13^ with Parkinson's disease in a cohort of 68 adults with 22q11.2 deletion syndrome aged 35 years and older. The patients had no family history of Parkinson's disease, genetic screening did not identify any known pathogenic mutations related to Parkinson's disease, and post-mortem analysis of three of four patients revealed classic loss of midbrain dopaminergic neurons, with two having α-synuclein-positive Lewy bodies. Together, these data suggest that microdeletions at 22q11.2 might confer an increased risk of Parkinson's disease.

These previous studies focused on identification of Parkinson's disease in carriers of the 22q11.2 deletion. In this study, we set out to establish the frequency of deletions spanning the 22q11.2 locus in idiopathic Parkinson's disease.

## Methods

### Study design and participants

We used data from four independent genome-wide association studies of Parkinson's disease that had been analysed for copy number variants (CNVs). The details of all samples from the four studies—namely, the UK Wellcome Trust Case Control Consortium 2 (WTCCC2) Parkinson's disease, Dutch Parkinson's Disease Genetics Consortium, US National Institute on Aging (NIA), and International Parkinson's Disease Genomics Consortium (IPDGC), have been described previously.^3^, ^4^, ^5^, ^6^, ^7^, ^8^ Most cases were recruited using UK Brain Bank Criteria, with modification in the IPDGC NeuroX study^15^ to allow patients with family history to be included, and for one subset (contributed by Academic Medical Center Amsterdam) of the Dutch cohort, the criteria by Gelb and colleagues were used.^16^ The US NIA controls were from the Neurogenetic Repository at the Coriell Institute for Medical Research, which excluded people with schizophrenia and other neurological diseases (appendix A); unscreened population controls were used in the other studies. Only patients and controls of European descent and unrelated were included.

The original studies^3^, ^4^, ^5^, ^6^, ^7^, ^8^ received approval from the relevant ethics committees, as previously published. Patients and controls or their legal representatives provided informed consent before recruitment..

### Procedures

Samples were genotyped using the Illumina 660,^3^, ^4^, ^5^, ^8^ Illumina 1·2M Duo,^3^, ^4^, ^5^ Illumina550,^6^ Illumina240S and Illumina317K,^6^ or the NeuroX arrays^7^ (Illumina, San Diego, CA, USA). We did CNV analysis for all autosomes using standard protocols, as described previously.^17^ Briefly, CNVs (minimum of ten single nucleotide polymorphisms) were identified using PennCNV (2011Jun16 version) and included GC content as a covariate in the PennCNV statistical model.^18^ Derivative of LogR ratio (LRR; LRR_SD) and derivative of B-allele frequency (BAF; BAF_SD) were generated by PennCNV; samples with an LRR_SD greater than 0·30, a BAF_SD greater than 0·15, or a number of CNVs 3 SDs above the mean CNV number of the case or control groups were excluded. Because some large CNVs can be erroneously split by CNV identifying algorithms, we merged CNVs larger than 100 kb that occurred in an individual if the gap was less than 50% of the entire length of the newly merged CNV. LRR and BAF of the newly created CNV were visually inspected before acceptance for inclusion.

The reported region typically deleted in chromosome 22q11.2 deletion syndrome is usually 3 Mb and spans from around 18·8 Mb to 21·8 Mb (hg19) on chromosome 22 or, less commonly, is 1·5 Mb (around 18·9 Mb to 20·4 Mb) and at the centromeric part of the 3 Mb region.^10^ To ensure that our CNV selection strategy identified atypical deletions at this locus, we selected all deletions identified in the cases and controls that spanned the region encompassed by the 1·5 Mb deletion by at least 60%. To validate the deletions, we used a customised array (CytoSure 15k-Array, Agilent-based Array Comparative Genomic Hybridisation, Oxford Gene Technology, Oxford, UK).

### Statistical analysis

We compared the proportions of chromosome 22q11.2 deletions between cases with Parkinson's disease and controls from four independent Parkinson's disease case-control studies. For each independent case-control study, we compared the two groups using the Fisher's exact test (two sided). For the meta-analysis of the four studies (ten studies when we split the IPDGC study into seven subgroups), we used the Mantel-Haenszel exact test (two sided) to avoid the Simpson's paradox.^19^, ^20^ A classic description of Simpson's paradox is when a pattern that appears in different groups of data disappears or reverses when these groups are combined. This situation can occur because of other hidden confounding variables not controlled by simple combination, leading to a paradoxical result. We also tested the contribution of each independent study to our meta-analysis by doing a sensitivity analysis, in which we sequentially excluded each independent study before repeating the meta-analysis. Rare mutations that confer an increased risk of Parkinson's disease occur typically in patients with Parkinson's disease with an earlier age at onset.^21^ To investigate the relation between 22q11.2 deletions and age at onset of disease, we compared the distribution of age at onset between deletion carriers and non-carriers with a Mann-Whitney *U* test. Patients are generally regarded as having early-onset Parkinson's disease if they have an age at onset younger than 45 years.^22^ The proportion of 22q11.2 deletions found in patients with early-onset and late-onset Parkinson's disease was compared with proportion in controls and also between patients with early-onset and late-onset Parkinson's disease using Fisher exact test and Mantel-Haenszel exact test. To estimate the odds ratio of Parkinson's disease in 22q11.2 deletion carriers, we assumed a population prevalence of 22q11.2 deletions of 0·024%,^23^ which is close to 25 in 100 000 livebirths.^9^ One-sided p value was also added in footnotes of result tables to enable readers who wish to compare with some previous CNV studies using one-sided test.

We did all statistical analyses in SPSS (version 20.0.0) and R (version 3.12). p values of 0·05 or lower were judged to be statistically significant.

### Role of the funding source

The funders of the study had no role in study design, data collection, data analysis, data interpretation, or writing of the report. KYM, US, NMW, and NWW had full access to all the data in the study and the corresponding authors had final responsibility for the decision to submit for publication.

## Results

9387 patients with Parkinson's disease and 13 863 controls were included from the four case-control studies. The disease age at onset was available for 8451 (90%) of 9387 patients with Parkinson's disease. Age at onset data were available for all patients carrying the 22q11.2 deletion.

The most common deletion associated with chromosome 22q11.2 deletion syndrome spans about 3 Mb (around hg19 chromosome 22: 18·8–21·8 Mb) and is flanked by LCRs A–D (figure). We identified eight unrelated patients with Parkinson's disease who carried the most common chromosome 22q11.2 deletion syndrome (3 Mb) deletion from three independent Parkinson's disease studies, with none detected in the fourth, smallest study (US NIA; figure; table 1). Using the customised CytoSure 15k-Array, we validated all the 3 Mb deletions in the UK groups (UK 1 and UK 2, data not shown). These data confirmed the deletion breakpoint findings from the Illumina array. We did not replicate the validation in other studies because the validation in UK samples showed convincing results using the Illumina array. Analysis of the largest independent study (UK WTCCC2, assuming the IPDGC NeuroX study is a meta-analysis of studies) revealed a pattern of enrichment of 22q11.2 deletion in patients with Parkinson's disease (p=0·059; table 1). A similar pattern was noted when the IPDGC NeuroX study was classed as a homogeneous study, using the Fisher's exact test (table 1). In the combined meta-analyses of all independent samples (9387 patients with Parkinson's disease and 13 863 controls; table 1), 22q11.2 deletions were associated with Parkinson's disease, both when the IPDCG NeuroX study was classed as one subgroup (four-subgroup analysis p=0·00056) and when each group within that study was classed as a separate subgroup (ten-subgroup analysis; p=0·00082). Although we only identified 3 Mb deletions and did not identify any of the infrequent but well-characterised 1·5 Mb deletions that typically span LCRs A–B, in a post-hoc inspection of CNVs at this locus, we identified one patient carrying an atypical 726 kb deletion spanning LCRs B–D (UK 3; figure). Association data were also significant after inclusion of this rare deletion (four-subgroup analysis p=0·00014; ten-subgroup analysis p=0·00020; appendix); however, because we were uncertain of the relation between this rare deletion and chromosome 22q11.2 deletion syndrome, we excluded it from our statistical analyses. Because the UK 3 deletion was not considered a chromosome 22q11.2 deletion syndrome, DNA was not sent for replication in comparative genomic hybridisation. Other than the 22q11.2 deletion, no other recurrent CNVs of moderate to large size (>500 kb) were found in these eight patients.

Findings from the sensitivity analysis revealed that association with deletions at 22q11.2 remained significant after the systematic exclusion of each independent study from the meta-analysis (all p≤0·016; appendix).

In the 8451 patients for whom age at onset data were available (table 2), age at onset of Parkinson's disease was lower in patients carrying a 22q11.2 deletion (median 37 years, 95% CI 32·0–55·5; mean 42·1 years [SD 11·9]) than in those who did not carry a deletion (median 61 years, 95% CI 60·5–61·0; mean 60·3 years [SD 12·8]; p=0·001). Patients are generally regarded as having early-onset Parkinson's disease if they have an age at onset younger than 45 years.^24^ Of the 1014 patients with Parkinson's disease with age at onset younger than 45 years, five (0·49%) had 22q11.2 deletions, compared with three (0·04%) of 7437 with an age at onset of 45 years or older (late-onset Parkinson's disease; four-subgroup analysis p=0·0007; ten-subgroup analysis p=0·005; table 3). A 22q11.2 deletion was present in more patients with early-onset (ten-subgroup analysis p<0·0001) and late-onset Parkinson's disease (p=0·016) than in controls (table 3). The findings were similar when we defined early-onset Parkinson's disease as onset younger than 50 years post-hoc (appendix A). The frequency of 22q11.2 deletions in controls (0%) is lower than the expected population frequency of 0·024% that was reported from a study of large cohorts.^21^ To address the possible bias, we estimated that at the population frequency of 0·024%, we expected to detect around three deletions at 22q11.2 in our control sample of 13 863 people. Association analysis revealed that, even when using this conservative estimate, the excess of 22q11.2 deletions in patients with early-onset Parkinson's disease was significant (p<0·0001; appendix).

With an estimated one in four carriers of the 22q11.2 deletion with schizophrenia, having a hemizygous deletion at 22q11.2 is one of the strongest known risk factors for development of the disease.^25^ Moreover, although in a large study the proportion of patients with schizophrenia who had a 22q11.2 deletion was 0·29%,^26^ studies using community catchment populations have yielded a proportion of 0·98%,^27^ and findings from a review suggested a proportion of up to 2·4%.^25^ In comparison, in the present study, we found that 0·49% of patients with idiopathic early-onset Parkinson's disease had 22q11.2 deletions. Post hoc, we attempted an estimation of increased risk of early-onset Parkinson's disease, conferred by deletions at 22q11.2, which is a 20-times increase compared with the general population (assuming the proportion of carriers of a 22q11.2 deletion is 0·024%; appendix A). However, in view of the small number of cases (five) and the large CIs, this finding should be considered an estimate of the conferred risk.

Table 4 describes the clinical features of the patients with Parkinson's disease who had a 22q11.2 deletion. The eight patients fulfilled the UK Brain Bank Criteria for Parkinson's disease, had asymmetry in onset of motor symptoms and tremor (seven of seven patients with assessment records available), developed bradykinesia and rigidity during the disease course (six of six patients with available data), and most had a good initial response to levodopa or dopamine agonist treatment (five of six). No structural abnormalities were noted in all three patients for whom MRI scan data were available (IPDGC NeuroX 4 and 5, and UK 1). Dopamine transporter scans were abnormal in all four patients for whom these data were available, with asymmetric deficits in two (UK 1 and Dutch 1) and a symmetrical decrease in the other two (IPDGC NeuroX 4 and 5), who had longer disease duration. None of the six patients for whom more detailed clinical histories were available had interrupted aortic arch type B, truncus arteriosus, or tetralogy of Fallot. Patient IPDGC NeuroX 3 had a valvular heart lesion, with no detailed information on the nature of cardiac anomaly. Mitral valve prolapse was discovered after Parkinson's disease was diagnosed in patient IPDGC NeuroX 5. Three patients had depression before diagnosis of Parkinson's disease (Dutch 1, UK 2, and IPDGC NeuroX 4: NeuroX4 might be reactive depression). None of the eight patients had schizophrenia diagnosed before onset of Parkinson's disease. Two patients had cleft palate or oropharyngeal malformation diagnosed before onset of Parkinson's disease (IPDGC NeuroX 1 and 5, respectively) and one was found to have bifid uvula during investigations for dysphagia after diagnosis of Parkinson's disease. IPDGC NeuroX 4 had hypocalcaemia. Two of the eight patients died at a young age (IPDGC NeuroX 2 and UK 2, at 51 and 56 years old respectively).

So far, no data exist on the prevalence of chromosome 22q11.2 deletion syndrome in Parkinson's disease. The prevalence of early-onset Parkinson's disease had been quoted to be about 3–5% of all Parkinson's disease cases.^28^ In the Cardiff community survey,^29^, ^30^ the estimated prevalence of Parkinson's disease was 140 per 100 000 people, with 3·6% of patients developing the disease before age 45 years, which is comparable to the 4·2% reported in Canada.^29^, ^30^ Assuming 4% of all patients with Parkinson's disease have early-onset disease and since about 0·5% had a 22q11.2 deletion in the present study, we estimate the prevalence of early-onset Parkinson's disease with a 22q11.2 deletion to be 0·028 per 100 000 people (140 × 4% × 0·5%).

## Discussion

In this study, we provide evidence that 22q11.2 deletions are associated with an increased risk of Parkinson's disease. In a previous observational study, an increased frequency of Parkinson's disease was reported in patients with chromosome 22q11.2 deletion syndrome compared with controls.^14^ Herein, we report the results of the reverse experiment, namely pooling of data from large Parkinson's disease case-control studies and assessment of the frequency of 22q11.2 deletion carriers. We found a significantly higher frequency of 22q11.2 deletions in both patients with early-onset and late-onset Parkinson's disease compared with controls, and in patients with early-onset compared with late-onset Parkinson's disease. Findings from the sensitivity analysis remained significant after the systematic exclusion of each independent study from the meta-analysis, which suggests that, despite the variation in sample size across studies, there was no evidence that the association was generated by a single study. We therefore suggest that presence of a 22q11.2 deletion is a risk factor for Parkinson's disease, in particular early-onset Parkinson's disease.

None of the patients identified presented with a diagnosis of chromosome 22q11.2 deletion syndrome. With hindsight, some cases had other features suggestive of 22q11.2 deletion syndrome, such as hypocalcaemia, depression, fatigue, mental retardation, and cleft palate. However, cleft palate (prevalence up to one in 700)^31^ and childhood seizures (prevalence 2–4%)^32^ are not rare. Depression occurs in 40% of patients with Parkinson's disease and is one of the non-motor symptoms.^33^ Apathy is found in 20–36% of patients with newly diagnosed Parkinson's disease and can pre-date Parkinson's disease.^34^ Therefore, these features would not necessarily be diagnostic for chromosome 22q11.2 deletion syndrome. One patient had a valvular heart lesion, but no detailed information was available. None of the six patients for whom more detailed clinical histories were available had characteristic cardiac features (interrupted aortic arch type B, truncus arteriosus, and Tetralogy of Fallout), which have been reported in over 60% of patients with chromosome 22q11.2 deletion syndrome.^35^ One patient had mitral valve prolapse, but this is not associated with chromosome 22q11.2 deletion syndrome. The aforementioned cardiac features might be insufficient to alert clinicians to the presence of 22q11.2 deletion in the Parkinson's disease patients.

An obvious question is whether the eight identified patients are typical cases of Parkinson's disease. Without pathological confirmation of Parkinson's disease, we cannot categorically exclude this possibility. However, the patients in this study fulfilled the UK Brain Bank Criteria for Parkinson's disease and had typical signs and symptoms of the disease. These include asymmetric onset of motor symptoms, and tremor, bradykinesia, and rigidity during the disease course. Additionally, most patients had a good initial response to levodopa or dopamine agonist treatment. The dopamine transporter scans available were comparable with those from patients with Parkinson's disease. All these findings are suggestive of Parkinson's disease in these patients. The early age at onset, early drug-related dyskinesia and motor fluctuations, and cognitive and psychotic features associated with treatment might provide clinical clues to the presence of chromosome 22q11.2 deletion syndrome. Any additional history of orofacial anomalies or cognitive or psychiatric illness in patients with Parkinson's disease with an early age of onset should also prompt clinicians to consider the possibility of chromosome 22q11.2 deletion.

The findings from this study are important for patient management in early-onset Parkinson's disease and insight into pathogenic mechanisms. In the investigation of early-onset Parkinson's disease, the possibility of a 22q11.2 deletion should be considered. Although the estimated prevalence among patients with early-onset Parkinson's disease was not high (0·49%), the presence of a 22q11.2 deletion has direct implications for management, especially the identification and medical management of comorbidities. Detailed guidelines on counselling for patients with 22q11.2 deletion syndrome have been reviewed.^36^, ^37^, ^38^ A few features specific to chromosome 22q11.2 deletion syndrome in Parkinson's disease are worth mentioning: genetic counselling should be provided to offspring (>90% of chromosome 22q11.2 deletion syndrome cases are de novo) and appropriate guidance given for prenatal testing, if needed; and cognitive issues need to be considered in terms of managing the patient's direct health care, counselling, and the implications that carrying a genetic defect (22q11.2 deletion) might have on family members. Management of comorbidities will need the input of specialist teams.

Two patients died at a young age (<60 years old). Two studies have assessed mortality in children with chromosome 22q11.2 deletion syndrome^39^, ^40^ and only one has investigated mortality and sudden death among adults.^41^ By adding paediatric and adult mortality from previous studies,^39^, ^40^, ^41^ we crudely estimate survival to be around 75% at 40 years old and less than 65% at 50 years old. We were not able to establish the cause of death in the two patients who died at a young age. None of the eight patients with Parkinson's disease and chromosome 22q11.2 deletion had a known history of major cardiac abnormality or schizophrenia. Most of the cases in the reported adult sudden death study had either a major cardiac abnormality or schizophrenia.^41^ Hence, the prognosis of the patients with Parkinson's disease and chromosome 22q11.2 deletion might be different from that of the patients in the sudden death study. Nevertheless, clinicians should still consider a cardiac work-up and monitor for the possibility of sudden death in patients with early-onset Parkinson's disease who carry the 22q11.2 deletion.

We note that this higher mortality in patients with chromosome 22q11.2 deletion syndrome compared with the general population^39^, ^40^, ^41^ might affect the assumed carrier frequency in the general population. If the actual population carrier frequency is lower than 0·024% (assumed frequency) because of the increased mortality, then our statistical significance would be more robust for the difference between the deletion carrier rate in Parkinson's disease detected versus the general population.

Chromosome 22q11.2 deletion syndrome arises in an estimated 25 in 100 000 livebirths,^9^ and our estimated prevalence of early-onset Parkinson's disease with a 22q11.2 deletion is 0·028 per 100 000 people. By assessing the clinical features of our cases and reviewing the published work, we have come up with potential explanations for this difference. First, none of the cases in this study were suspected to have chromosome 22q11.2 deletion syndrome and none have had schizophrenia or a severe congenital heart anomaly, both of which are common in people with chromosome 22q11.2 deletion syndrome. Our recruitment of patients with Parkinson's disease must have excluded most patients with chromosome 22q11.2 deletion syndrome with characteristic severe cardiac disorders or schizophrenia, even though this was not a planned exclusion criteria. Second, three of eight patients had age of onset of Parkinson's disease between 45 years and 60 years old, which is outside our early-onset Parkinson's disease cutoff age. These two factors probably resulted in a substantial underestimate of the prevalence of a 22q11.2 deletion in early-onset Parkinson's disease. Mortality is high in children with chromosome 22q11.2 deletion syndrome^39^, ^40^ and also in carriers surviving into adulthood.^41^ In a report by Butcher,^14^ few patients with chromosome 22q11.2 deletion syndrome developed Parkinson's disease, which suggests that other important modifying factors have a role in the penetrance of Parkinson's disease among chromosome 22q11.2 deletion syndrome. Last, but not least, early-onset Parkinson's disease is uncommon. Underestimation of the prevalence of 22q11.2 deletion among people with Parkinson's disease, increased mortality of 22q11.2 deletion carriers, and non-penetrance of 22q11.2 deletion contribute to the discrepancy between the prevalence of chromosome 22q11.2 deletion syndrome overall and in people with early-onset Parkinson's disease who carry the 22q11.2 deletion.

Drug-induced dyskinesia and cognitive changes in people with Parkinson's disease who have the 22q11.2 deletion might be related to the underlying pathogenic mechanism (eg, perhaps related to a change in catechol-*O*-methyltransferase [COMT] concentration secondary to loss of one copy of the *COMT* gene located within the chromosome 22q11.2 region). Alternatively, it might be resulted from a combination of early age at onset of Parkinson's disease, disease duration, and *COMT* deficiency.

How heterozygous microdeletions on chromosome 22q11.2 lead to the diverse spectrum of associated phenotypes is not well understood. Therefore, the pathogenic mechanisms that confer an increased risk of Parkinson's disease warrant particular attention. Parkinson's disease is probably caused by haploinsufficiency of one or more dose-sensitive genes within the deleted region. A second, as yet unidentified, recessive mutation in the remaining hemizygous allele within 22q11.2 might also contribute to Parkinson's disease. Finally, a 22q11.2 deletion might result in alteration of position effects.^42^

So far, probably because of the paucity of appropriate tissue samples, the effect of hemizygosity on gene expression in neural tissue from patients with chromosome 22q11.2 deletion syndrome has not been investigated. Instead, gene expression has been investigated in blood cells using expression arrays and these have shown that many genes spanned by deletions at 22q11.2 have reduced expression in deletion carriers.^43^, ^44^ Located within the 1·5 Mb region deleted in 22q11DS, the *COMT* gene is a strong candidate for Parkinson's disease because it encodes an enzyme that degrades catecholamines, including dopamine, which is therapeutically relevant to Parkinson's disease. Although Parkinson's disease is not regarded as a disorder of dopamine metabolism, the identification that *GCH1* mutations can lead to Parkinson's disease as well as dopa-responsive dystonia suggests that biochemical depletion of dopamine is likely to have more complicated effects than once thought.^45^ Findings from expression array studies using blood cells^43^, ^44^ showed decreased *COMT* mRNA expression among patients with chromosome 22q11.2 deletion syndrome compared with controls. However, this finding cannot be directly equated to decreased enzymatic activity of COMT. The activity in the brain is further complicated by the presence of the membrane-bound COMT isoform. In a meta-analysis of a well-characterised functional non-synonymous COMT polymorphism, rs4680 (Val158Met),^46^ no evidence was found for an increased risk of Parkinson's disease. Therefore, further work is needed to assess comprehensively the possible relation between hemizygosity of 22q11.2 deletion, long-term COMT deficiency, and clinical presentations in chromosome 22q11.2 deletion syndrome.

In our analysis, we excluded a patient who carried the atypical 726 kb deletion (patient UK 3) because the deletion in that narrow region was not reported to be associated with chromosome 22q11.2 deletion syndrome. However, we cannot exclude the possibility that this deletion could suggest a minimal region of relevance for Parkinson's disease (ie, the smallest genomic region in which a deletion leads to development of Parkinson's disease).

This study has several limitations. We did a retrospective combined analysis of four studies, which could be non-homogeneous. The methods of case referral might also have resulted in an ascertainment bias. A history of schizoaffective disorder was not an explicit exclusion criterion in the recruitment of patients with Parkinson's disease, and although it is not part of the UK Brain Bank exclusion criteria, the UK Brain Bank does exclude patients having neuroleptic treatment at the onset of symptoms of Parkinson's disease. Moreover, since 22q11.2 deletion carriers with severe psychiatric or other systemic illness, or both, would probably be managed by a psychiatrist and other specialists, they are less likely to have been referred to neurology recruitment centres and therefore are more likely to have been excluded from the study. This ascertainment bias would have lowered the percentage of 22q11.2 deletion carriers in the Parkinson's disease cohorts. Thus, in this study, 0·49% of patients with early-onset Parkinson's disease probably represents the lower limit of the prevalence of 22q11.2 deletions in early-onset Parkinson's disease.

We also recognise that variation in recruitment criteria of the control groups could have affected the low rate of 22q11.2 deletion carriers identified. For example, although unselected population controls were used in the UK sample, people with schizophrenia were excluded from the Coriell controls in the US sample. Therefore, this exclusion might have resulted in a decreased frequency of 22q11.2 deletions in controls and an artificial increase in the association. Based on an estimated prevalence of schizophrenia of 3·3% in a meta-analysis of 188 cohorts,^47^ and an estimated 1% prevalence of chromosome 22q11.2 deletion syndrome among people with schizophrenia (meta-analysis and community data),^25^, ^27^ less than one case of chromosome 22q11.2 deletion syndrome (0·24) would be detected in the Coriell controls (n=726) if schizophrenia was not excluded. Therefore, we believe that the effect of excluding people with schizophrenia in the Coriell controls had little effect on our results and is unlikely to have affected the final conclusion.

Additionally, participation of psychiatric patients might have been reduced in the control groups in this study, which could have resulted in an artificially reduced prevalence of 22q11.2 deletions. However, our finding was still significant when we made the comparison with the reported population frequency. In view of the high early mortality in chromosome 22q11.2 deletion syndrome (around 25% before age 40 years, as mentioned earlier), our population frequency of 0·024% might have been an overestimate and the p value might therefore have been underestimated.

A further limitation is that the four genome-wide association studies were cross-sectional studies, rather than prospective studies, and we could only retrospectively trace the clinical details from medical records of the deletion carriers. Not all of the patients had received long-term follow-up or had been assessed for progression systematically. Moreover, we did not have access to comprehensive clinical data and cannot conclusively exclude the possibility of the use of neuroleptics before referral. Therefore, although we cannot rule out the possibility that treatment of psychiatric symptoms in some 22q11.2 deletion carriers could have led to the development of drug-induced parkinsonism, we are not aware of any of the eight deletion carriers having a severe psychiatric illness before ascertainment, and in view of the positive dopamine transporter scans for the four available patients and the positive response to levodopa or dopamine agonists for five of six patients, we believe that this is unlikely. Because of scarce detailed clinical data, we confined our analyses to simple, but robust, phenotypes (eg, age at onset).

In conclusion, we provide evidence that deletions at 22q11.2 increase the risk of Parkinson's disease, particularly the early-onset form. In view of the strong association between the well-characterised 22q11.2 deletion and schizophrenia, clinicians should be aware of the possibility that a 22q11.2 deletion might underlie Parkinson's disease with early presentation, psychiatric features, or other associated features, or a combination thereof. Understanding the mechanisms behind this dichotomy could give important insights into both developmental neurophysiology and disease pathogenesis. The precise biological mechanisms and whether additional genetic or epigenetic factors play a part in the increased risk of Parkinson's disease among 22q11.2 deletion carriers should be addressed in future studies.

## Figures and Tables

**Figure:**
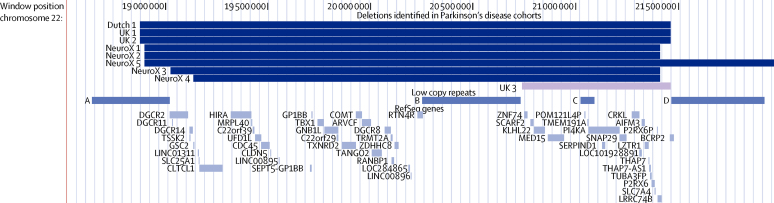
Genomic location of the 22q11.2 deletions found in the eight patients with Parkinson's disease and location of the low copy repeat regions UK3 represented an atypical deletion of uncertain relation to 22q11.2 deletion syndrome and was not considered here. Figure drawn using the UCSC Genome Browser.^24^

**Table 1:** Association studies of deletions at chromosome 22q11.2

		**Patients with Parkinson's disease**		**Controls**		**p value**	
		Number of patients	Number with 22q11.2 deletion (%)	Number	Number with 22q11.2 deletion	Two-sided Fisher's exact test	Two-sided Mantel-Haenszel test
**Studies**							
US NIA		593	0	726	0	1·00	..
UK WTCCC2		1592	2 (0·13%)	4939	0	0·059	..
Dutch Parkinson's Disease Genetics Consortium		740	1 (0·14%)	1996	0	0·271	..
IPDGC NeuroX		6462	5 (0·08%)	6202	0	0·063*	0·069†
	UK	804	2 (0·25%)	684	0	0·50	..
	USA	2069	0	2652	0	1·00	..
	France	564	2 (0·35%)	479	0	0·50	..
	Germany	1298	1 (0·08%)	883	0	1·00	..
	Greece	736	0	891	0	1·00	..
	Netherlands	316	0	447	0	1·00	..
	PPMI or other	675	0	166	0	1·00	..
**Meta-analyses**							
Four subgroups (IPDGC NeuroX counted as one subgroup)		9387	8	13 863	0	..	0·00056
Ten subgroups (each group in IPDGC NeuroX counted separately)		9387	8	13 863	0	..	0·00082

p values for the one-sided Fisher's exact tests were the same as for the two-sided tests, apart from for IPDGC NeuroX, which was p=0·035. p values for the one-sided Mantel-Haenszel tests were the same as for the two-sided tests. IPDGC=International Parkinson's Disease Genomics Consortium. NIA=National Institute on Aging. PPMI=Parkinson's Progression Marker Initiative. WTCCC2=Wellcome Trust Case Control Consortium 2.

* Assuming the populations were non-heterogeneous and adding the number of cases or controls together.

† Treating IPDGC NeuroX as a seven-subgroup meta-analysis.

**Table 2:** Patients with Parkinson's disease entered into the age at onset analysis

	**Patients with Parkinson's disease**			**Patients with Parkinson's disease and known age of onset**		
	Total	With 22q11.2 deletion				
		Male	Female	Number (%)*	Male	Female
US NIA	593	0	0	593 (100%)	355	238
UK WTCCC2	1592	1	1	1545 (97%)	900	645
Dutch Parkinson's Disease Genetics Consortium	740	1	0	714 (96%)	456	258
IPDGC NeuroX	6462	4	1	5599 (87%)	3584	2015
Total	9387	6	2	8451 (90%)	5295	3156

IPDGC=International Parkinson's Disease Genomics Consortium. NIA=National Institute on Aging. WTCCC2=Wellcome Trust Case Control Consortium 2.

* Percentage of total number of patients with Parkinson's disease.

**Table 3:** Comparison of deletions at 22q11.2 according to Parkinson's disease age at onset

		**Patients with EOPD (age of onset <45 years)**		**Patients with LOPD (age of onset ≥45 years)**		**Total**	**Controls**		**Association test p values**		
		Number of patients	Number with 22q11.2 deletion (%)	Number of patients	Number with 22q11.2 deletion (%)		Number of controls	Number with 22q11.2 deletion (%)	EOPD *vs* LOPD	EOPD *vs* controls	LOPD *vs* controls
**Studies**											
US NIA		121	0	472	0	593	726	0	1·0*	1·0*	1·0*
UK WTCCC2		94	1 (1·06%)	1451	1 (0·07%)	1545	4939	0	0·12*	0·019*	0·23*
Dutch Parkinson's Disease Genetics Consortium		139	0	575	1 (0·17%)	714	1996	0	1·0*	1·0*	0·22*
IPDGC NeuroX		660	4 (0·61%)	4939	1 (0·02%)	5599	6202	0	0·0009*†	<0·0001*†	0·443*†
	UK	131	1 (0·76%)	320	1 (0·31%)	451	684	0	0·50*	0·16*	0·32*
	USA	131	0	1858	0	1989	2652	0	1·0*	1·0*	1·0*
	France	108	2 (1·9%)	455	0	563	479	0	0·037*	0·034*	1·0*
	Germany	177	1 (0·56%)	962	0	1139	883	0	0·16*	0·17*	1·0*
	Greece	46	0	642	0	688	891	0	1·0*	1·0*	1·0*
	Netherlands	16	0	81	0	97	447	0	1·0*	1·0*	1·0*
	PPMI or other	51	0	621	0	672	166	0	1·0*	1·0*	1·0*
**Meta-analyses**											
Four subgroups (IPDGC NeuroX counted as one subgroup)		1014	5	7437	3	8451	13 863	0	0·0007‡	<0·0001‡	0·023‡
Ten subgroups (each group in IPDGC NeuroX counted separately)		1014	5	7437	3	8451	13 863	0	0·005‡	<0·0001‡	0·016‡

p values for one-sided tests were the same as for the two-sided tests. EOPD=early-onset Parkinson's disease. IPDGC=International Parkinson's Disease Genomics Consortium. LOPD=late-onset Parkinson's disease. NIA=National Institute on Aging. PPMI=Parkinson's Progression Marker Initiative. WTCCC2=Wellcome Trust Case Control Consortium 2.

* Two-sided Fisher's exact test.

† Assuming the populations were non-heterogeneous and adding the number of cases and controls together.

‡ Two-sided Mantel-Haenszel test.

**Table 4:** Clinical features of the patients with Parkinson's disease carrying 22q11.2 deletions

	**Clinical features at presentation**	**Other neurological features**	**Treatment and complication**	**Progression and follow-up**	**Psychiatric/cognitive problem after pd diagnosis**	**Imaging (MRI or DAT)**	**Genetics**	**Past psychiatric, cognitive, or congenital medical history**
NeuroX_1	Initial presentation: pain and then tremor in left arm	Nil mentioned	Good response to L-dopa	Rigidity, bradykinesia, and gait abnormality with freezing at year 7; dysarthria and dysphagia; EEG normal	Psychiatric symptoms with depressive episode, optical delusions as well as anxiety and self-injured behaviour; loss to follow-up before imaging	NA	Negative PARK2 (Parkin) and PINK1; NeuroX array includes most of the known Parkinson Mendelian mutations	Nil psychiatric problems reported by patient, cleft palate surgery at 8, congenital strabismus
NeuroX_2	NA	NA	No detail available	No detail available; dyskinesia within 10 year of onset; died at 51 years old	NA	NA	Negative PARK2 (Parkin); NeuroX array includes most of the known Parkinson Mendelian mutations	NA
NeuroX_3	Right side tremor, worse with anxiety; difficult writing, occasional difficult feeding	Nil mentioned	Madopar and doapmine agonist, scaling up	Assessment at year 6 : hypomimia, dysarthric speech, and hypophonia; flexed posture; bilateral tremor, right side much more affected than left side; mark rigidity especially the neck; retropulsion; difficulty in writing; worked up for deep brain stimulation; while pending workup, deteriorated in swallowing and pharyngeal pouch found; last seen at year 8 with severe tremor in both arms, moderate rigid-hypokinetic, short steps, shuffling; no freezing, balance impaired; loss to follow-up afterward	Nil in year 6 and 7 for psychiatric symptoms; psychometric test: low average range on verbal and borderline-impaired on performance	NA	NeuroX array includes most of the known Parkinson Mendelian mutations	Nil psychiatric problems reported by patient; bifid uvula (found in assessment for dysphagia, after Parkinson's disease diagnosis), tinnitus, hernia; valvular heart, right shoulder replacement
NeuroX_4	Akinesia, rigidity, and tremor in right hand	Nil mentioned	Good initial response to dopamine agonist, then add on levodopa after 5 years	Motor fluctuations and dyskinesia 5 years after onset; at year 8 typical levodopa induced dyskinesia, peak dose chorea; dysarthria, dysphonia; no gait impairment, no falls	At year 8, mild cognitive impairment with dysexecutive syndrome	MRI: normal; DAT: severe bilateral presynaptic denervation	NeuroX array includes most of the known Parkinson Mendelian mutations	Depression (reactive, after death of spouse 10 year before onset of Parkinson's disease); cataract; hypocalcaemia (1·8mmol/L), parathyroid hormone (PTH) 3·3 pg/mL (N 12–65); no reported dysmorphia; appendectomy
NeuroX_5	Intermittent tremor of left upper limb	Nil mentioned	Dopamine agonist and then levodopa (started after 2 years) with good initial response; levodopa-induced dyskinesia (1 year after levodopa started)	At year 3 akinesia and extrapyramidal rigidity of the limbs, bilateral, asymmetric left more than right plus pyramidal syndrome of the four limbs and bilateral postural tremor; freezing at year 4; gait impairment at year 7; swallowing problem with gastrostomy at year 7; Pneumonia at year 4	Confusion with levodopa at 650 mg/day	MRI failed; DAT scan (at year 5): bilateral dopaminergic denervation	NeuroX array includes most of the known Parkinson Mendelain mutations	Mental and language retardation, congenital oro-pharyngeal malformation (hypotrophy), mitral valve prolapse (diagnosed after Parkinson's disease onset)
UK_1	Difficulty using left hand due to slowness, stiffness, and intermittent tremor; examination showed hypomimic, flex postures and left side rigidity	Nil mentioned	Positive response on L-dopa challenge; dopamine agonists, maobis, and apomorphine were not tolerated; confusion with tolcapone; on duodopa	Early motor fluctuations, painful dystonic off	Florid psychosis, visual hallucinations and delusion with escalating treatment	MRI: left frontal periventricular cysts of unknown significance and a partially empty sella; DAT scan: bilateral deficits presynaptic dopamine uptake in the striata most severe in the putamina	Recruitment criteria included exclusion of known genes at study [included PARK2 (Parkin), PINK1]	Learning difficulties, delayed motor milestones, mastoidectomy for ear infection, infant seizures till age 4 years
UK_2	Tremor and slow in walking; examination confirmed reduced right arm swing, right arm tremor, tremulous but not micrographic writing	Twitching of lips, tremulous voice, tremor improved with alcohol	No improvement with madopar; improvement with propanolol, procyclidine	Died 3 years after first assessment; record not available to confirm the cause and no detail about the progress available	NA	NA	Recruitment criteria included exclusion of known genes at study [included PARK2 (Parkin), PINK1]	Depression
Dutch_1	Resting tremor left leg and left arm, slow in walking	Nil mentioned	NA	Lost to follow-up after 1 year	NA	No MRI abnormalities; DAT scan: decreased uptake in putamen, right side more affected than left side	NA	Chronic fatigue 18 years before Parkinson's disease diagnosis, and depression at least more than 3 years before diagnosis, no known congenital anomaly
UK_3 (not counted as 22q11.2 deletion)	NA	NA	NA	NA	NA	NA	Recruitment criteria included exclusion of known genes at study [included PARK2 (Parkin), PINK1]	NA

All patients with deletion had an age at onset younger than 60 years old, earlier than the median of 61 years (95% 60·5–61·0), and mean of 60·3 years (SD 12·8) in those without deletions. Median age at onset of the patients with deletion was 37 years (95% CI 32·0–55·5) and mean was 42·1 years (SD 11·9). Five of eight patients had age at onset lower than 45 years old, fulfil our definition of early-onset Parkinson's disease. All deletion cases were not labelled with 22q11·2DS. Seven of eight cases had information on onset. All were asymmetric at onset; had tremor at onset, bradykinesia and rigidity, consistent with the UK Brain Bank Criteria. Five of six patients had initial good response to dopaminergic treatment, be it with dopamine agonists or L-dopa therapy. Dopamine Transporter imaging (DAT Scan) was available for four cases; all showed presynaptic deficits in striatal uptake, with scans asymmetric in two compatible with asymmetric onset and symmetrical decrease in the other two with longer disease duration. Five of five patients with long-term follow-up information showed some psychiatric/cognitive complications during the treatment period; two had hallucination and psychosis, one with confusion on levodopa 650 mg/day, and two with cognitive impairment. Patients with Parkinson's disease with 22q11·2 deletion seemed to have deteriorated much faster and had severe drug related dyskinesia within a few years; this contrast slower progress for early-onset Parkinson's disease in general. Two (25%) of eight patients were documented dead; we were not able to establish the cause of death. Male-to-female ratio was 6:2 in cases with 22qDel vs Parkinson's disease without 22qDel (5289:3154); no significant difference, Fisher-exact test p=0·72. No detail information on the atypical deletion (UK-3) for comparison. The UK study protocol had the known gene mutations excluded at the time of study (this included PARK2 (Parkin) and PINK1). No data were reported in Dutch study. The US-NIA study had the early onset Parkinson's disease checked for PARK2 (Parkin) and PINK1. In NeuroX, most of the known Parkinson's disease mutations were analysed as they were included on the genotyping array, with some limitations in small deletion.
