# Supplementary material for: Deletions at 22q11.2 in idiopathic Parkinson's disease: a combined analysis of genome-wide association data
Source: Lancet Neurol. 2016 May;15(6):585–96. doi: 10.1016/S1474-4422(16)00071-5 (PMC4828586; doi:10.1016/S1474-4422(16)00071-5)
Supplement: Supplementary appendix [file mmc1.pdf]

THE LANCET Neurology

Supplementary webappendix

This webappendix formed part of the original submission and has been peer reviewed. We post it as supplied by the authors.

Supplement to: Mok KY, Sheerin U, Simón-Sánchez J, et al. Deletions at 22q11.2 in idiopathic Parkinson's disease: a combined analysis of genome-wide association data. *Lancet Neurol* 2016; published online March 23. http://dx.doi.org/10.1016/S1474-4422(16)00071-5.

IPDGC consortium members and affiliations: Mike A Nalls (Laboratory of Neurogenetics, National Institute on Aging, National Institutes of Health, Bethesda, MD, USA), Vincent Plagnol (UCL Genetics Institute, London, UK), Dena G Hernandez (Laboratory of Neurogenetics, National Institute on Aging; and Department of Molecular Neuroscience, UCL Institute of Neurology, London, UK), Manu Sharma (Department for Neurodegenerative Diseases, Hertie Institute for Clinical Brain Research, University of Tübingen, and DZNE, German Center for Neurodegenerative Diseases, Tübingen, Germany), Una-Marie Sheerin (Department of Molecular Neuroscience, UCL Institute of Neurology), Mohamad Saad (INSERM U563, CPTP, Toulouse, France; and Paul Sabatier University, Toulouse, France), Javier Simón-Sánchez (Department for Neurodegenerative Diseases, Hertie Institute for Clinical Brain Research, University of Tübingen, and DZNE, German Center for Neurodegenerative Diseases, Tübingen, Germany), Claudia Schulte (Department for Neurodegenerative Diseases, Hertie Institute for Clinical Brain Research), Suzanne Lesage (INSERM, UMR_S975 [formerly UMR_S679], Paris, France; Université Pierre et Marie Curie-Paris, Centre de Recherche de l'Institut du Cerveau et de la Moelle épinière, Paris, France; and CNRS, Paris, France), Sigurlaug Sveinbjörnsdóttir (Department of Neurology, Landspítali University Hospital, Reykjavík, Iceland; Department of Neurology, MEHT Broomfield Hospital, Chelmsford, Essex, UK; and Queen Mary College, University of London, London, UK), Sampath Arepalli (Laboratory of Neurogenetics, National Institute on Aging), Roger Barker (Department of Neurology, Addenbrooke's Hospital, University of Cambridge, Cambridge, UK), Yoav Ben-Shlomo (School of Social and Community Medicine, University of Bristol), Henk W Berendse (Department of Neurology and Alzheimer Center, VU University Medical Center), Daniela Berg (Department for Neurodegenerative Diseases, Hertie Institute for Clinical Brain Research and DZNE, German Center for Neurodegenerative diseases), Kailash Bhatia (Department of Motor Neuroscience, UCL Institute of Neurology), Rob M A de Bie (Department of Neurology, Academic Medical Center, University of Amsterdam, Amsterdam, Netherlands), Alessandro Biffi (Center for Human Genetic Research and Department of Neurology, Massachusetts General Hospital, Boston, MA, USA; and Program in Medical and Population Genetics, Broad Institute, Cambridge, MA, USA), Bas Bloem (Department of Neurology, Radboud University Nijmegen Medical Centre, Nijmegen, Netherlands), Zoltan Bochdanovits (Department of Clinical Genetics, Section of Medical Genomics, VU University Medical Centre), Michael Bonin (Department of Medical Genetics, Institute of Human Genetics, University of Tübingen, Tübingen, Germany), Jose M Bras (Department of Molecular Neuroscience, UCL Institute of Neurology), Kathrin Brockmann (Department for Neurodegenerative Diseases, Hertie Institute for Clinical Brain Research and DZNE, German Center for Neurodegenerative diseases), Janet Brooks (Laboratory of Neurogenetics, National Institute on Aging), David J Burn (Newcastle University Clinical Ageing Research Unit, Campus for Ageing and Vitality, Newcastle upon Tyne, UK), Elisa Majounie (Laboratory of Neurogenetics, National Institute on Aging), Gavin Charlesworth (Department of Molecular Neuroscience, UCL Institute of Neurology), Codrin Lungu (National Institutes of Health Parkinson Clinic, NINDS, National Institutes of Health), Honglei Chen (Epidemiology Branch, National Institute of Environmental Health Sciences, National Institutes of Health, NC, USA), Patrick F Chinnery (Neurology M4104, The Medical School, Framlington Place, Newcastle upon Tyne, UK), Sean Chong (Laboratory of Neurogenetics, National Institute on Aging), Carl E Clarke (School of Clinical and Experimental Medicine, University of Birmingham,

Birmingham, UK; and Department of Neurology, City Hospital, Sandwell and West Birmingham Hospitals NHS Trust, Birmingham, UK), Mark R Cookson (Laboratory of Neurogenetics, National Institute on Aging), J Mark Cooper (Department of Clinical Neurosciences, UCL Institute of Neurology), Jean Christophe Corvol (INSERM, UMR_S975; Université Pierre et Marie Curie-Paris; CNRS; and INSERM CIC-9503, Hôpital Pitié-Salpêtrière, Paris, France), Carl Counsell (University of Aberdeen, Division of Applied Health Sciences, Population Health Section, Aberdeen, UK), Philippe Damier (CHU Nantes, CIC0004, Service de Neurologie, Nantes, France), Jean-François Dartigues (INSERM U897, Université Victor Segalen, Bordeaux, France), Panos Deloukas (Wellcome Trust Sanger Institute, Wellcome Trust Genome Campus, Cambridge, UK), Günther Deuschl (Klinik für Neurologie, Universitätsklinikum Schleswig-Holstein, Campus Kiel, Christian-Albrechts-Universität Kiel, Kiel, Germany), David T Dexter (Parkinson's Disease Research Group, Faculty of Medicine, Imperial College London, London, UK), Karin D van Dijk (Department of Neurology and Alzheimer Center, VU University Medical Center), Allissa Dillman (Laboratory of Neurogenetics, National Institute on Aging), Frank Durif (Service de Neurologie, Hôpital Gabriel Montpied, Clermont-Ferrand, France), Alexandra Dürr (INSERM, UMR_S975; Université Pierre et Marie Curie-Paris; CNRS; and AP-HP, Pitié-Salpêtrière Hospital), Sarah Edkins (Wellcome Trust Sanger Institute), Jonathan R Evans (Cambridge Centre for Brain Repair, Cambridge, UK), Thomas Foltynie (UCL Institute of Neurology), Jing Dong (Epidemiology Branch, National Institute of Environmental Health Sciences), Michelle Gardner (Department of Molecular Neuroscience, UCL Institute of Neurology), J Raphael Gibbs (Laboratory of Neurogenetics, National Institute on Aging; and Department of Molecular Neuroscience, UCL Institute of Neurology), Alison Goate (Department of Psychiatry, Department of Neurology, Washington University School of Medicine, MI, USA), Emma Gray (Wellcome Trust Sanger Institute), Rita Guerreiro (Department of Molecular Neuroscience, UCL Institute of Neurology), Clare Harris (University of Aberdeen), Jacobus J van Hilten (Department of Neurology, Leiden University Medical Center, Leiden, Netherlands), Albert Hofman (Department of Epidemiology, Erasmus University Medical Center, Rotterdam, Netherlands), Albert Hollenbeck (AARP, Washington DC, USA), Janice Holton (Queen Square Brain Bank for Neurological Disorders, UCL Institute of Neurology), Michele Hu (Department of Clinical Neurology, John Radcliffe Hospital, Oxford, UK), Xuemei Huang (Departments of Neurology, Radiology, Neurosurgery, Pharmacology, Kinesiology, and Bioengineering, Pennsylvania State University–Milton S Hershey Medical Center, Hershey, PA, USA), Isabel Wurster (Department for Neurodegenerative Diseases, Hertie Institute for Clinical Brain Research and German Center for Neurodegenerative diseases), Walter Mätzler (Department for Neurodegenerative Diseases, Hertie Institute for Clinical Brain Research and German Center for Neurodegenerative diseases), Gavin Hudson (Neurology M4104, The Medical School, Newcastle upon Tyne, UK), Sarah E Hunt (Wellcome Trust Sanger Institute), Johanna Huttenlocher (deCODE genetics), Thomas Illig (Institute of Epidemiology, Helmholtz Zentrum München, German Research Centre for Environmental Health, Neuherberg, Germany), Pálmi V Jónsson (Department of Geriatrics, Landspítali University Hospital, Reykjavík, Iceland), Jean-Charles Lambert (INSERM U744, Lille, France; and Institut Pasteur de Lille, Université de Lille Nord, Lille, France), Cordelia Langford (Cambridge Centre for Brain Repair), Andrew Lees (Queen Square Brain Bank for Neurological Disorders), Peter Lichtner (Institute of Human Genetics, Helmholtz Zentrum München, German Research

Centre for Environmental Health, Neuherberg, Germany), Patricia Limousin (Institute of Neurology, Sobell Department, Unit of Functional Neurosurgery, London, UK), Grisel Lopez (Section on Molecular Neurogenetics, Medical Genetics Branch, NHGRI, National Institutes of Health), Delia Lorenz (Klinik für Neurologie, Universitätsklinikum Schleswig-Holstein), Codrin Lungu (National Institutes of Health Parkinson Clinic, NINDS, National Institutes of Health), Alisdair McNeill (Department of Clinical Neurosciences, UCL Institute of Neurology), Catriona Moorby (School of Clinical and Experimental Medicine, University of Birmingham), Matthew Moore (Laboratory of Neurogenetics, National Institute on Aging), Huw R Morris (National Hospital for Neurology and Neurosurgery, University College London, London, UK), Karen E Morrison (School of Clinical and Experimental Medicine, University of Birmingham; and Neurosciences Department, Queen Elizabeth Hospital, University Hospitals Birmingham NHS Foundation Trust, Birmingham, UK), Valentina Escott-Price (MRC Centre for Neuropsychiatric Genetics and Genomics, Cardiff University School of Medicine, Cardiff, UK), Ese Mudanohwo (Neurogenetics Unit, UCL Institute of Neurology and National Hospital for Neurology and Neurosurgery), Sean S O'Sullivan (Queen Square Brain Bank for Neurological Disorders), Justin Pearson (MRC Centre for Neuropsychiatric Genetics and Genomics), Joel S Perlmutter (Department of Neurology, Radiology, and Neurobiology at Washington University, St Louis), Hjörvar Pétursson (deCODE genetics; and Department of Medical Genetics, Institute of Human Genetics, University of Tübingen), Pierre Pollak (Service de Neurologie, CHU de Grenoble, Grenoble, France), Bart Post (Department of Neurology, Radboud University Nijmegen Medical Centre), Simon Potter (Wellcome Trust Sanger Institute), Bernard Ravina (Translational Neurology, Biogen Idec, MA, USA), Tamas Revesz (Queen Square Brain Bank for Neurological Disorders), Olaf Riess (Department of Medical Genetics, Institute of Human Genetics, University of Tübingen), Fernando Rivadeneira (Departments of Epidemiology and Internal Medicine, Erasmus University Medical Center), Patrizia Rizzu (Department of Clinical Genetics, Section of Medical Genomics, VU University Medical Centre), Mina Ryten (Department of Molecular Neuroscience, UCL Institute of Neurology), Stephen Sawcer (University of Cambridge, Department of Clinical Neurosciences, Addenbrooke's hospital, Cambridge, UK), Anthony Schapira (Department of Clinical Neurosciences, UCL Institute of Neurology), Hans Scheffer (Department of Human Genetics, Radboud University Nijmegen Medical Centre, Nijmegen, Netherlands), Karen Shaw (Queen Square Brain Bank for Neurological Disorders), Ira Shoulson (Department of Neurology, University of Rochester, Rochester, NY, USA), Joshua Shulman (Baylor College of Medicine, Houston, Texas), Ellen Sidransky (Section on Molecular Neurogenetics, Medical Genetics Branch, NHGRI), Colin Smith (Department of Pathology, University of Edinburgh, Edinburgh, UK), Chris C A Spencer (Wellcome Trust Centre for Human Genetics, Oxford, UK), Hreinn Stefánsson (deCODE genetics), Francesco Bettella (deCODE genetics), Joanna D Stockton (School of Clinical and Experimental Medicine), Amy Strange (Wellcome Trust Centre for Human Genetics), Kevin Talbot (University of Oxford, Department of Clinical Neurology, John Radcliffe Hospital, Oxford, UK), Carlie M Tanner (Clinical Research Department, The Parkinson's Institute and Clinical Center, Sunnyvale, CA, USA), Avazeh Tashakkori-Ghanbaria (Wellcome Trust Sanger Institute), François Tison (Service de Neurologie, Hôpital Haut-Lévêque, Pessac, France), Daniah Trabzuni (Department of Molecular Neuroscience, UCL Institute of Neurology), Bryan J Traynor (Laboratory of Neurogenetics, National Institute on Aging), André G Uitterlinden (Departments of

Epidemiology and Internal Medicine, Erasmus University Medical Center), Daan Velseboer (Department of Neurology, Academic Medical Center), Marie Vidailhet (INSERM, UMR_S975, Université Pierre et Marie Curie-Paris, CNRS, UMR 7225), Robert Walker (Department of Pathology, University of Edinburgh), Bart van de Warrenburg (Department of Neurology, Radboud University Nijmegen Medical Centre), Mirdhu Wickremaratchi (Department of Neurology, Cardiff University, Cardiff, UK), Nigel Williams (MRC Centre for Neuropsychiatric Genetics and Genomics), Caroline H Williams-Gray (Department of Neurology, Addenbrooke's Hospital), Sophie Winder-Rhodes (Department of Psychiatry and Medical Research Council and Wellcome Trust Behavioural and Clinical Neurosciences Institute, University of Cambridge), Kári Stefánsson (deCODE genetics), Maria Martinez (INSERM UMR 1043; and Paul Sabatier University), Nicholas W Wood (UCL Genetics Institute; and Department of Molecular Neuroscience, UCL Institute of Neurology), John Hardy (Department of Molecular Neuroscience, UCL Institute of Neurology), Peter Heutink (DZNE, German Center for Neurodegenerative Diseases and Department for Neurodegenerative Diseases, Hertie Institute for Clinical Brain Research, University of Tübingen, Tübingen, Germany), Alexis Brice (INSERM, UMR_S975, Université Pierre et Marie Curie-Paris, CNRS, UMR 7225, AP-HP, Pitié-Salpêtrière Hospital), Thomas Gasser (Department for Neurodegenerative Diseases, Hertie Institute for Clinical Brain Research, and DZNE, German Center for Neurodegenerative Diseases), Andrew B Singleton (Laboratory of Neurogenetics, National Institute on Aging).

ACKNOWLEDGMENTS

We would like to thank all of the subjects who donated their time and biological samples to be a part of this study.

This work was supported in part by the Intramural Research Programs of the National Institute of Neurological Disorders and Stroke (NINDS), the National Institute on Aging (NIA), and the National Institute of Environmental Health Sciences both part of the National Institutes of Health, Department of Health and Human Services; project numbers Z01-AG000949-02 and Z01-ES101986. In addition this work was supported by the Department of Defense (award W81XWH-09-2-0128), and The Michael J Fox Foundation for Parkinson's Research. This work was supported by National Institutes of Health grants R01NS037167, R01CA141668, P50NS071674, American Parkinson Disease Association (APDA); Barnes Jewish Hospital Foundation; Greater St Louis Chapter of the APDA; Hersenstichting Nederland; Neuroscience Campus Amsterdam; and the section of medical genomics, the Prinses Beatrix Fonds. The KORA (Cooperative Research in the Region of Augsburg) research platform was started and financed by the Forschungszentrum für Umwelt und Gesundheit, which is funded by the German Federal Ministry of Education, Science, Research, and Technology and by the State of Bavaria. This study was also funded by the German National Genome Network (NGFNplus number 01GS08134, German Ministry for Education and Research); by the German Federal Ministry of Education and Research (NGFN 01GR0468, PopGen); and 01EW0908 in the frame of ERA-NET NEURON and Helmholtz Alliance Mental Health in an Ageing Society (HA-215), which was funded by the

Initiative and Networking Fund of the Helmholtz Association. The French GWAS work was supported by the French National Agency of Research (ANR-08-MNP-012). This study was also funded by France-Parkinson Association, the French program “Investissements d’avenir” funding (ANR-10-IAIHU-06) and a grant from Assistance Publique-Hôpitaux de Paris (PHRC, AOR-08010) for the French clinical data. This study was also sponsored by the Landspitali University Hospital Research Fund (grant to SSv); Icelandic Research Council (grant to SSv); and European Community Framework Programme 7, People Programme, and IAPP on novel genetic and phenotypic markers of Parkinson’s disease and Essential Tremor (MarkMD), contract number PIAP-GA-2008-230596 MarkMD (to HP and JHu). This study utilized the high-performance computational capabilities of the Biowulf Linux cluster at the National Institutes of Health, Bethesda, Md. (http://biowulf.nih.gov), and DNA panels, samples, and clinical data from the National Institute of Neurological Disorders and Stroke Human Genetics Resource Center DNA and Cell Line Repository. People who contributed samples are acknowledged in descriptions of every panel on the repository website.

We thank the French Parkinson’s Disease Genetics Study Group and the Drug Interaction with genes (DIGPD) study group. We also thank the members of the French 3C Consortium for allowing us to use part of the 3C cohort, and for support in generating the genome-wide molecular data. We thank Molecular Neurology Programme, Biomedicum, University of Helsinki, Department of Neurology, Helsinki University Central Hospital, Folkhalsan Institute of Genetics and Department of Pathology, University of Helsinki, and Department of Public Health and General Practice Division of Geriatrics, University of Eastern Finland for the Finnish controls (Vantaa85+ GWAS data).

We used genome-wide association data generated by the Wellcome Trust Case-Control Consortium 2 (WTCCC2) from UK patients with Parkinson’s disease and UK control individuals from the 1958 Birth Cohort and National Blood Service. Genotyping of UK replication cases on ImmunoChip was part of the WTCCC2 project, which was funded by the Wellcome Trust (083948/Z/07/Z). UK population control data was made available through WTCCC1. This study was supported by the Medical Research Council and Wellcome Trust disease centre (grant WT089698/Z/09/Z to NW, JHa, and ASc). As with previous IPDGC efforts, this study makes use of data generated by the Wellcome Trust Case-Control Consortium. A full list of the investigators who contributed to the generation of the data is available from www.wtccc.org.uk. Funding for the project was provided by the Wellcome Trust under award 076113, 085475 and 090355. This study was also supported by Parkinson’s UK (grants 8047 and J-0804) and the Medical Research Council (G0700943). DNA extraction work that was done in the UK was undertaken at University College London Hospitals, University College London, who received a proportion of funding from the Department of Health’s National Institute for Health Research Biomedical Research Centres funding. This study was supported in part by the Wellcome Trust/Medical Research Council Joint Call in Neurodegeneration award (WT089698) to the Parkinson’s Disease Consortium (UKPDC), whose members are from the UCL Institute of Neurology, University of Sheffield, and the Medical Research Council Protein Phosphorylation Unit at the University of Dundee.

Supplemental Table 1: Recruitment criteria of the four cohorts used

Studies	case				control			
	criteria	gender (M:F)	Age	reference	criteria	gender (M:F)	Age	reference
**USA-NIA**	All patients were diagnosed according to the UK Brain Bank criteria	1.49:1	Mean Age at onset: 56.6<br>SD: 13.9<br>Mean Age at sampling :NA	http://www.nature.com/ng/journal/v41/n12/full/ng.487.html	All individuals are reported to be unrelated Caucasians free from any neurological disorders. All individuals were asked specifically regarding the following disorders: Alzheimer's disease, amyotrophic lateral sclerosis, ataxia, autism, bipolar disorder, cerebrovascular disease, dementia, dystonia, Parkinson's disease, and schizophrenia.	0.71:1	Age range 15-98 (mean age ~58)	http://www.nature.com/ng/journal/v41/n12/full/ng.487.html
**UK_WTCCC2**	Study cases were collected from 5 UK centres, meeting the UK Brain Bank Clinical Criteria for PD	1.40:1	Mean Age at onset: 62.3<br>SD: 12.2<br>Mean Age at sampling :NA	http://hmg.oxfordjournals.org/cgi/pmidlookup?view=long&pmid=21044948	population controls. Controls from WTCCC2 comprised of a) 1958 birth cohort (1958C) collected at age 44-45; b) blood donor in UK Blood Services (NBS) age 18-69 .	M:F 1.06 (1958C)<br>M:F 0.99 (NBS)	Age at sampling: a) 1958C<br>44 (range 44-45)<br>Age at sampling: b) NBS (range 18-69)	http://www.nature.com/nature/journal/v447/n7145/full/nature05911.html
**The Dutch PD Genetics consortium**	All patients were diagnosed according to the UK Brain Bank criteria, except 1 contributing	1.75:1	Mean Age at onset: 55.1<br>SD: 11.9<br>Mean Age	http://www.nature.com/ejhg/journal/v19/n6/full/ejhg2010254a.html<br>http://archneur.j	Population controls from the Rotterdam study III	0.78:1	Age at sampling: 53.8 (range 45-95)	http://www.nature.com/ejhg/journal/v19/n6/full/ejhg2010254a.html

	centre, Academic Medical Center Amsterdam, using criteria proposed by Gelb et al		at sampling :62	amanetwork.com/article.aspx?articleid=774675				http://link.springer.com/article/10.1007/s10654-009-9386-z/fulltext.html
**NeuroX (IPDGC)**	all cases were recruited from clinic visit; standard UK Brain Bank criteria with a modification to allow the inclusion of cases that had a family history of PD	1.78:1	Mean Age at onset: 60.2 SD: 12.5 Mean Age at sampling :NA	http://www.nature.com/ng/journal/v46/n9/full/ng.3043.html suppl table 1	recruited from clinic visit and self-reporting	1.21:1	Mean age 63.2 (SD15.6)	http://www.nature.com/ng/journal/v46/n9/full/ng.3043.html suppl table 1

Supplemental Table 2: Association Study of Deletions at 22q11.2 PLUS atypical deletion with Parkinson's disease and Comparison Subjects.

		PD Subjects				Comparison Subjects				
**Studies**		**22q11.2 Deletion (N)**	**No Deletion (N)**	**Total (N)**	**Frequency of Deletion (%)**	**22q11.2 Deletion (N)**	**No Deletion (N)**	**Frequency of deletion (%)**	**Fishers Exact P-value**	**Mantel-Haenszel Exact P value**
USA-NIA		0	593	593	0	0	726	0·00	1·00 '	
UK-WTCCC2		3	1589	1592	0·19	0	4939	0·00	0·014 '	
Dutch		1	739	740	0·14	0	1996	0·00	0·271 '	
IPDGC_NeuroX	Sub-groups									
IPDGC	UK	2	802	804	0·25	0	684	0·00	0·50 '	
IPDGC	US	0	2069	2069	0·00	0	2652	0·00	1·00 '	
IPDGC	French	2	562	564	0·35	0	479	0·00	0·50 '	
IPDGC	German	1	1297	1298	0·08	0	883	0·00	1·00 '	
IPDGC	Greek	0	736	736	0·00	0	891	0·00	1·00 '	
IPDGC	Dutch	0	316	316	0·00	0	447	0·00	1·00 '	
IPDGC	PPMI/other	0	675	675	0·00	0	166	0·00	1·00 '	
IPDGC_NeuroX	all	5	6457	6462	0·08	0	6202	0·00	0·063 ' [a]	0.069 * [b]
Meta-analysis of All Studies #		8	9379	9387	-	0	13863	-	-	0·00014 * [c]
**Meta-analysis of All Studies ~**		8	9379	9387	-	0	13863	-	-	0·00020 * [d]

* Mantel-Haenszel Exact Test (2-sided), ' Fishers Exact Test (2-sided)

p value for 1-sided Fishers Exact Test same as 2-sided except IPDGC 1 sided test is 0.035

p value for 1-sided Mantel-Haenszel Exact Test on Meta-analysis of All Studies same as 2-sided, except IPDGC_NeuroX 1-sided is 0.051

a Fishers Exact test (2-sided) assuming the populations are non-heterogeneous and adding the number of cases or controls together

b Mantel-Haenszel Exact Test (2-sided) for the IPDGC_NeuroX, treating IPDGC_NeuroX as 7 subgroups metaanalysis

c Mantel-Haenszel Exact Test (2-sided) assuming the IPDGC_NeuroX as a single study

d Mantel-Haenszel Exact Test (2-sided) treating the IPDGC_NeuroX as 7 different studies plus US-NIA, UK-WTCCC2 and Dutch, a total of 10 studies

USA-NIA, UK-WTCCC2, Dutch, and IPDGC_NeuroX 4 groups metaanalysis

~ USA-NIA, UK-WTCCC2, Dutch, IPDGC_NeuroX_UK, IPDGC_NeuroX_US, IPDGC_NeuroX_French, IPDGC_NeuroX_German, IPDGC_NeuroX_Greek, IPDGC_NeuroX_Dutch, and IPDGC_NeuroX_PPMI 10 groups metaanalysis

Supplemental Table 3: Sensitivity analysis of all studies

Tests treating IPDGC_NeuroX as a single study

Excluded USA_NIA	PD Subjects			Comparison Subjects			
**Studies**	**22q11.2 Deletion (N)**	**No Deletion (N)**	**Frequency (%)**	**22q11.2 Deletion (N)**	**No Deletion (N)**	**Frequency (%)**	**Mantel-Haenszel test P-value**
UK-WTCCC2	2	1590	0·13	0	4939	0·00	
Dutch	1	739	0·14	0	1996	0·00	
IPDGC_NeuroX	5	6457	0·08	0	6202	0·00	
**Meta-analysis of All Studies**	8	8786	-	0	13137	-	0·00056

Excluded UK-WTCCC2	PD Subjects	-	-	Comparison Subjects	-	-	
**Studies**	**22q11.2 Deletion (N)**	**No Deletion (N)**	**Frequency (%)**	**22q11.2 Deletion (N)**	**No Deletion (N)**	**Frequency (%)**	**Mantel-Haenszel test P-value**
USA-NIA	0	593	0	0	726	0·00	
Dutch	1	739	0·13	0	1996	0·00	
IPDGC_NeuroX	5	6457	0·08	0	6202	0·00	
**Meta-analysis of All Studies**	6	7789	-	0	8924	-	0·0093

Excluded Dutch	PD Subjects	-	-	Comparison Subjects	-	-	
**Studies**	**22q11.2 Deletion (N)**	**No Deletion (N)**	**Frequency (%)**	**22q11.2 Deletion (N)**	**No Deletion (N)**	**Frequency (%)**	**Mantel-Haenszel test P-value**
USA-NIA	0	593	0	0	726	0·00	
UK-WTCCC2	2	1590	0·13	0	4939	0·00	
IPDGC_NeuroX	5	6457	0·08	0	6202	0·00	
**Meta-analysis of All Studies**	7	8640	-	0	11867	-	0·0020

Excluded IPDGC_NeuroX<br>Studies	PD Subjects<br>22q11.2 Deletion (N)	-<br>No Deletion (N)	-<br>Frequency (%)	Comparison Subjects<br>22q11.2 Deletion (N)	-<br>No Deletion (N)	-<br>Frequency (%)	Mantel-Haenszel test P-value
USA-NIA	0	593	0	0	726	0·00	
UK-WTCCC2	2	1590	0·13	0	4939	0·00	
Dutch	1	739	0·14	0	1996	0·00	
**Meta-analysis of All Studies**	3	2922	-	0	7661	-	0·016

All association tests were performed using the 2-sided Mantel-Haenszel Exact Test (p values of 2-sided test equal to 1-sided)

Supplemental Table 4: Comparison of Deletions at 22q11.2 according to Parkinson’s disease Age at Onset (<50 or >50 years old)

		Frequency of 22q11.2 deletions [N CNVs/N samples]										Association Tests (P-value)		
		EOPD Subjects (AAO<50years)				LOPD Subjects (AAO>=50years)				Total PD case with known AAO	Controls % [No. of deletion /total controls]			
		22q Del (N)	No 22qDel (N)	total (N)	% with deletion	22q Del (N)	No 22qDel (N)	total (N)	% with deletion			EOPD vs LOPD	EOPD vs Controls	LOPD vs Controls
**Studies**														
USA-NIA		0	168	168	0·00	0	425	425	0·00	593	0% [0/726]	1·0 '	1·0 '	1·0 '
UK-WTCCC2		1	181	182	0·55	1	1362	1363	0·07	1545	0% [0/4939]	0·222 '	0·036 '	0·216 '
Dutch		0	237	237	0·00	1	476	477	0·21	714	0% [0/1996]	1·0 '	1·0 '	0·193 '
IPDGC_NeuroX	Sub-groups													
IPDGC_NeuroX	UK	1	187	188	0·53	1	262	263	0·38	451	0% [0/684]	1·0 '	0·22 '	0·28 '
IPDGC_NeuroX	US	0	271	271	0·00	0	1718	1718	0·00	1989	0% [0/2652]	1·0 '	1·0 '	1·0 '
IPDGC_NeuroX	FRENCH	2	183	185	1.08	0	378	378	0·00	563	0% [0/479]	0·11 '	0·077 '	1·0 '
IPDGC_NeuroX	GERMAN	1	274	275	0·36	0	864	864	0·00	1139	0% [0/883]	0·24 '	0·24 '	1·0 '
IPDGC_NeuroX	GREEK	0	86	86	0·00	0	602	602	0·00	688	0% [0/891]	1·0 '	1·0 '	1·0 '
IPDGC_NeuroX	DUTCH	0	29	29	0·00	0	68	68	0·00	97	0% [0/447]	1·0 '	1·0 '	1·0 '
IPDGC_NeuroX	PPMI/OTHER	0	102	102	0·00	0	570	570	0·00	672	0% [0/166]	1·0 '	1·0 '	1·0 '
IPDGC_NeuroX	All	4	1132	1136	0·35	1	4462	4463	0·02	5599	0% [0/6202]	7·07E-3 ' [a]	5·72E-4 ' [a]	0·419 ' [a]
Meta-analysis of All Studies #		5	1718	1723	-	3	6725	6728	-	8451	0% [0/13863]	8·92E-3*[b]	2·03E-5*[b]	0·017*[b]
**Meta-analysis of All Studies ~**		5	1718	1723	-	3	6725	6728	-	8451	0% [0/13863]	0·043*[c]	1·41E-4*[c]	0·012*[c]

Notes:

* Mantel-Haenszel Exact Test (2-sided), ' Fishers Exact Test (2-sided)

P values for Fishers Exact Test (2-sided) and Mantel-Haenszel Exact Test (2-sided) equal to 1-sided except IPDGC_UK EOPD vs LOPD with p=0·66 in 1-sided test;

a Fishers Exact test (2-sided) assuming the populations are non-heterogeneous and adding the number of cases or controls together

b Mantel-Haenszel Exact Test (2-sided) assuming the IPDGC_NeuroX as a single study

c Mantel-Haenszel Exact Test (2-sided) treating the IPDGC_NeuroX as 7 different studies plus US-NIA, UK-WTCCC2 and Dutch, a total of 10 studies

USA-NIA, UK-WTCCC2, Dutch, and IPDGC_NeuroX 4 groups metaanalysis

~ USA-NIA, UK-WTCCC2, Dutch, IPDGC_NeuroX_UK, IPDGC_NeuroX_US, IPDGC_NeuroX_French, IPDGC_NeuroX_German, IPDGC_NeuroX_Greek, IPDGC_NeuroX_Dutch, and IPDGC_NeuroX_PPMI 10 groups metaanalysis

A classic description of a Simpson's Paradox is when a trend that appears in different groups of data, disappears or reverses when these groups are combined. This can be due to other hidden confounding variables not controlled by simple combination leading to a paradoxical result

Supplemental Table 5: Association study between proportion of 22q11.2 deletion in EOPD vs control population (population frequency 0·024%)

Studies	Frequency of 22q11.2 deletions [N CNVs/N samples] EOPD Subjects (AAO<45years)	Controls (assumed rate)	EOPD vs Controls (p value)	OR (95% confidence interval)
**All Studies Combined**	0·49% [5/1014]	0·022% [3/13863]	6·9 E-5 '	22·9 (4·4 - 147)
**All Studies Combined**	0·49% [5/1014]	0·029% [4/13863]	1·5 E-4 '	17·2 (3·7 - 86)

' Fishers Exact Test (2-sided)
(1-sided test have same p values; 95% interval is 5·6 – infinity and 4·6 – infinity respectively)

We have attempted to calculate a stratified assessment of the O.R. in individual studies and meta-analysis assuming population frequency of 0·024% or roughly 1 in 4166.[21] However, there are practical issues if the frequency in control is low. For example, as the number of control subjects in most of the studies is smaller than 4000 the potential number of deletions found in the controls will be less than 1. This less than unity cannot have an O.R. and P-value calculated by Fisher Exact test. If we artificially increase the number for control to 1 in 4166, the p value for the O.R. will either be artificially inflated or decreased. Hence, stratified assessment of the O.R. is not done.

Our aim was to provide an estimate of the O.R. so that readers could compare the risk conferred by 22q11.2 deletions to EOPD with the well-established increased risk to schizophrenia (for which the published estimations of O.R. are also affected by the same limitations as those in this current study). For this reason, in this Supplemental Table 5, there are two estimated scenarios for the O.R. based on identifying 3 (0·022%) and 4 (0.029%) deletions in a hypothetical control population of around 13863 controls(equals to 3.3 carriers given prevalence of 0·024%). As stratification study is not feasible, we simply add the number of deletions in cases together.

Supplemental Table 6: Estimation of increased risk to develop Early Onset Parkinson's Disease in 22q11.2 deletion carrier

Assume population frequency of 22q11.2 deletion(22qDel) is 0·024%.[21]

	22qDel +ve	22qDel -ve	total
EOPD	5 (=a)	1009 (=b)	a+b
Control	c	d	c+d
Total	a+c	b+d	

Odds ratio = Odds of case in 22qDel positive / Odds of case in 22qDel negative

Odds of case in 22qDel positive = [a / (a+c)] / [c / (a+c)] = a / c

Odds of case in 22qDel negative = [b / (b+d)] / [d / (b+d)] = b / d

Thus Odds ratio is (a/c) / (b /d) = (a/b) / (c/d)

We do not know precisely the values of c and d but we know that c / (c+d) is the population frequency = 0·024%.[21]

We also know c is extremely rare and so as c is << d, then c / (c+d) is almost equivalent to c / d.

Thus, odds ratio in this setting is roughly = (a/b) / (c/d) = 5/1009 or 0·496% / 0·024%

Since the 22qDel frequency in EOPD as calculated is 0·493% [a/ (a+b) or 5 / (5+1009)]. To streamline and keep the calculation more simple in the main text, we used a / (a+b), i.e. 0·49% rather than a/b, i.e. 0·496%.

Thus the Odds ratio calculated is 0·49% / 0·024% = 20·4.

However, as stated in Supplemental table 5, the best way to calculate the OR is stratified studies, not adding the number of all studies together. However, as explained in Supplemental table 5, the deletion is relatively rare and making hypothetical deletions found in controls well below unity. This make a proper stratified meta-analysis of OR infeasible. Hence, a simple summation is used here in Supplemental table 5 and 6.
